# Estimating Individual and Household Reproduction Numbers in an Emerging Epidemic

**DOI:** 10.1371/journal.pone.0000758

**Published:** 2007-08-22

**Authors:** Christophe Fraser

**Affiliations:** Medical Research Council Centre for Outbreak Analysis and Modelling, Department of Infectious Disease Epidemiology, Imperial College London, London, United Kingdom; Yale University, United States of America

## Abstract

Reproduction numbers, defined as averages of the number of people infected by a typical case, play a central role in tracking infectious disease outbreaks. The aim of this paper is to develop methods for estimating reproduction numbers which are simple enough that they could be applied with limited data or in real time during an outbreak. I present a new estimator for the individual reproduction number, which describes the state of the epidemic at a point in time rather than tracking individuals over time, and discuss some potential benefits. Then, to capture more of the detail that micro-simulations have shown is important in outbreak dynamics, I analyse a model of transmission within and between households, and develop a method to estimate the household reproduction number, defined as the number of households infected by each infected household. This method is validated by numerical simulations of the spread of influenza and measles using historical data, and estimates are obtained for would-be emerging epidemics of these viruses. I argue that the household reproduction number is useful in assessing the impact of measures that target the household for isolation, quarantine, vaccination or prophylactic treatment, and measures such as social distancing and school or workplace closures which limit between-household transmission, all of which play a key role in current thinking on future infectious disease mitigation.

## Introduction

The household is a fundamental unit of transmission for many directly transmitted infections. In addition, the household provides a “laboratory” within which key measures of transmission such as infectiousness, generation time and the effect of immunity or vaccination can be studied [1]. In recent years considerable effort has gone into understanding the dynamics of transmission within populations organised into households using mathematical models [2], [3], [4], [5], [6]. Most effort has gone into analysing the asymptotic behaviour of these models, elucidating the threshold levels of transmission required for infection to be self-sustaining, calculating final epidemic sizes, or predicting the impact of generalised or targeted interventions designed to reduce or eliminate transmission. In parallel, methods have been derived to estimate the parameters which govern transmission within the household from detailed case reports [7], [8], [9], [10]. However, scant effort appears to have been paid to how to apply household structured models to the analysis of epidemics, either retrospectively or in real time.

Concurrently, mathematical models have played an ever greater role in interpreting and responding to emerging pathogens. These models have typically been either of the “simple but tractable” variety which ignore or average over demographic structure and social mixing patterns [11], [12] or the “complex computer simulation” variety that capture many details of demographic structure and dynamics, but of whom the behaviour can only be determined by intensive numerical analysis [13], [14], [15]. The aim of this study is to develop methods of a perhaps “slightly less simple but still tractable” variety that capture some of the detail that micro-simulations have shown is important, but which can be rapidly applied (say on a daily basis) in an emerging outbreak situation, to inform policy. More specifically, the aim is to arrive at a method to estimate the key transmission and control parameters for a model of transmission within and between households from as few detailed observations as are likely to be gathered in the heat of a major outbreak.

The resulting analysis will still be based on major simplifications in respect to all the spatial and other social constructs that govern disease transmission, but less so than those based on the very simplest assumption of free, homogeneous mixing. In this context, it should be stated that even in the best, most robustly parameterised microsimulations, gross approximations are made in describing the fabulously complex web of human behaviour, and even they are only attempts to characterise the statistical properties of the system as a whole. Extensive effort is, and should continue to be, spent on identifying the conditions where different types of simplification (household models, static network models, spatial metapopulation models…) can and can't be justified, and in developing analytical approximations to describe disease transmission within such simplified structures.

Individual based simulations of influenza and smallpox pandemic spread and control, incorporating detailed information on population density, age structure, commuting patterns, workplace sizes and long-distance travel have highlighted the particular importance of the household as a fundamental unit of transmission [13], [14], [16], [17], [18] (and reviewed in [19]). Pure household models have been used fruitfully to explore detailed policy options in a city-wide response to an influenza pandemic [20]. It thus seems *a priori* that household models are a natural starting point in terms of extending theory previously developed for the simplest assumption of homogeneous mixing.

The analysis presented here will focus on deriving new estimators for individual and household reproduction numbers, denoted *R* (*t* ) and *R* ^*^ (*t* ) respectively. The individual reproduction number *R* (*t* ) is defined roughly as the average number of people someone infected at time *t* can infect over their entire infectious lifespan; as I will show below, there are several ways of defining this more precisely. The household reproduction number R^*^ (*t* ) is defined here as the average number of households a household infected at time *t* can infect [3], [6]. The individual reproduction number *R* (*t* ) rightly plays a privileged role in epidemiology, as it is a meaningful measure within any contact network. However, of the possible summary measures of epidemic progress, it is not necessarily the most useful. For example, for an emerging directly transmitted pathogen, such as pandemic influenza virus, public health interventions may target the household rather than the individual, enforcing household quarantine as well as offering antivirals to the household to limit transmission within the household. In such a situation, the household reproduction number R ^*^ (*t* ) is more directly related to the parameters which characterize the intervention, and is thus a better measure of the effect of these interventions. These quantities (*R* (*t* ) and R ^*^ (*t* )) share the two essential properties of reproduction numbers, namely that they increase when infectiousness increases and decrease when infectiousness decreases (monotonicity), and that they mark a threshold that separates exponentially growing epidemics (when *R* (*t* )>1 or equivalently R ^*^ (*t* )>1) from exponentially declining epidemics (when *R* (*t* )<1 or equivalently R ^*^ (*t* )<1) [3], [6].

The structure of the paper focuses first on deriving estimators for individual reproduction numbers, then on household reproduction numbers and finally on examples of pandemic influenza dynamics and measles.

## Methods

### Estimating reproduction numbers using a Kermack McKendrick transmission model

Though less well known than their compartmental counterparts (SIR, SIS, etc…), time-since-infection models offer a more intuitive starting point for modelling infectious disease transmission, and importantly for this application, they provide two other major advantages. First, it is typically easier to identify their key parameters, and second they more readily adapt to describe multi-level transmission (by multi-level, I mean here within-household and between household). A disadvantage is that it can be harder to include heterogeneities. Nomenclature is confusing, since both types of model have their origin in the same classic paper of Kermack and McKendrick [21], and both the SIR model and the simplest time-since-infection model are known as “the Kermack-McKendrick model”.

The model, in the formalism chosen here, predicts the changing incidence rate *I* (*t*) as a function of calendar time *t* in terms of the transmissibility, denoted β (*t*, τ ), an arbitrary function of calendar time *t* and time since infection τ. β (*t*, τ) typically reflects pathogen load, or perhaps more precisely pathogen shedding. It is commonly a single peaked function reflecting pathogen growth followed by immune suppression, or host death, but can be more exotic such as the double peaked profile associated with early and late transmission of HIV [22], or the repeated peaks of malaria [23]. β (*t*, τ) also reflects the effective contact rate between infectious and susceptible individuals, which can change during the course of a single infection, increasing for example if a person coughs or sneezes due to respiratory disease, or decreasing if a person takes to bed with illness, and during the course of the epidemic as public health measures are implemented. More discussions of the components (infectiousness and contact) of β (*t*, τ) can be found in [24]. Because I am interested in outbreaks of emerging infections, I will not describe explicitly reductions in the susceptible population caused by the epidemic. Formally this corresponds to working in the infinite population limit. This assumption is not essential for this section however, since β (*t*, τ) could also be thought of as incorporating the proportion of cases that are susceptible; the assumption becomes more important in the later sections on household models.

Mathematically, transmission is defined by a Poisson infection process such that the probability that, between time *t* and *t*+δ, someone infected a time *t* ago successfully infects someone else is β (*t*, τ)δ, where δ is a very small time interval.

This assumption then results in a prediction that the mean incidence *I* (*t* ) at time *t* follows the so-called renewal equation
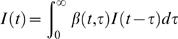
(1)


This equation states that the number of newly infected individuals is proportional to the number of prevalent cases multiplied by their infectiousness. It may often be convenient (and realistic) to truncate the function β (*t*, τ) at a time τ*_m_* such that β (*t*, τ) = 0 for all τ>τ_m_.

The asymptotic behaviour of incidence *I* (*t* ) is determined by reproduction numbers [21], [25]. Two intuitively defined reproduction numbers are the case reproduction number, which I denote *R_c_* (*t* ), and the instantaneous reproduction number, which I denote *R* (*t*). The case reproduction number *R_c_* (*t* ) is a property of individuals infected at time *t*, and is the average number of people someone infected at time *t* can expect to infect. For a person infected at time *t* it is the total infection hazard from time *t* onwards, i.e.
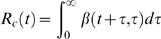
(2)


While the case reproduction number has been widely used, it may also be worth considering a quantity which I call the instantaneous reproduction number *R* (*t* ), a property of the epidemic at time *t*. It is the average number of people someone infected at time *t* could expect to infect should conditions remain unchanged. It is given by
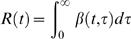
(3)


To illustrate the distinction between *R_c_* (*t* ) and *R* (*t* ), consider a situation where the transmission rate is abruptly reduced at a time *t* = *t_I_*. The instantaneous reproduction number *R* (*t* ), which estimates how many people one case would infect if circumstances were to remain fixed, would abruptly switch from a high to a low value at time *t_I_*. The case reproduction number *R_c_* (*t* ), on the other hand, estimates how many people each case actually infects. It will thus account for the fact that someone infected at time *t*<*t_I_* may spend part of their infectious period before and after the reduction in transmission which occurs at time *t_I_* and thus *R_c_* (*t* ) will smoothly transition from higher to lower values.

To derive simple estimating equations for *R* (*t* ), I consider the case where this function is separable, which corresponds to saying that the relative progression of infectiousness as a function of time since infection is independent of calendar time. In this case β (*t*, τ) can be written as the product of two functions Φ_1_ (*t* ) and Φ_2_ (τ), i.e.
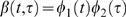
(4)


A counter-example might be when reactive patient isolation is introduced and acts to reduce infectiousness in late stage infection, in which case β (*t*, τ) can't be decomposed in this way. For this type of situation, it may be reasonable to assume the β (*t*, τ) can be decomposed separately in different stages of the epidemic, pre- and post- implementation of isolation measures, for example.

Since β (*t*, τ) is a product, I can arbitrary normalise one or other of the functions Φ_1 _(*t*) and Φ_2 _(τ), so without loss of generality, I choose Φ_2_ (τ) to have total integral 1, i.e. 
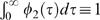
. Substituting equation (4) into equation (3) I find that
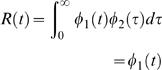
(5)


The function Φ_1_ (*t*) is equal to the instantaneous reproduction number *R* (*t*). The function Φ_2_ (τ) is then the distribution of how these infection events are distributed as a function of time since infection τ. This is an idealised definition of the generation time distribution, which I denote *w* (*t*). Thus, infectiousness can be decomposed as the product of the instantaneous reproduction number and the generation time distribution, i.e.
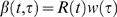
(6)


The relationship between the idealised generation time distribution *w* (τ) and the distribution of observed generation times can be rather complex for a number of reasons. First, infections are rarely observed, and thus must be either back-calculated or the generation times must be based on a surrogate such as the appearance of symptoms [1], [12]. Second, right censoring can cause the observed generation times to be shorter or longer than expected for a growing or declining epidemic, respectively [26]. Third, as apparent here, if the reproduction number *R* (*t*) changes due to depletion of susceptibles, changes in contact rates or public health measures, then this will also change the observed generation times for infectious individuals during that period of change. Thus the distribution *w* (τ) is really intended as a measure of infectiousness which will correspond to generation times for an index case in an ideal large closed setting where contact rates are constant. It can be inferred from data on the timing of cases, as in [10], [13].

Inserting (6) into (1) yields a novel estimator for instantaneous reproduction number
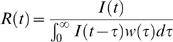
(7)


By substituting the decomposition (6) into equation (2), a relation between the instantaneous and case reproduction number is obtained:
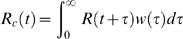
(8)i.e. the case reproduction number is a smoothed function of the instantaneous reproduction number.

Usually, incidence is reported as a discrete time series of the form *I_i_* incident cases reported between time *t_i_* and time *t_i_*+1, in which case the generation time distribution should be appropriately discretised into a form *w_i_* such that 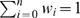. The estimators for the reproduction numbers become
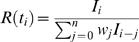
(9)and
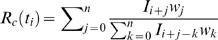
(10)


Equation (10) was proposed by [12], [27] as a real time estimator of the reproduction number, while equation (9) was first used for analysing polio transmission in India [28] (based on the work presented in this manuscript).

While the case reproduction number is an intuitively appealing quantity, the instantaneous reproduction number estimated by equation (9) should also be considered for practical applications as it may suffer fewer problems of right censoring in an incompletely observed epidemic. Right censoring is a real problem in using the case reproduction number to track an epidemic in real time, since the estimator for *R_c_* (*t*) at time *t* is seen in equation (10) to rely on knowing the incidence at future time-points. An algorithm to deal with this issue was proposed by [29], but switching instead to the instantaneous reproduction number estimated by equation (9) may be a simpler solution. Right censoring is not however the only complication associated with estimating reproduction numbers in practice, and is not completely absent from (7) due to the delay in detecting infections. Left censoring may also arise due to not knowing the baseline number infected if an epidemic has been unfolding for some time before observations are recorded. Finally, estimating the generation time distribution may not be straightforward.

Several strategies are possible to deal with the fact that one never observes infections, but rather as a time series of cases of the form *C_i_*, where case definitions could be based on symptoms, hospitalisation or seroconversion. One strategy, used in [12], is simply to ignore this and use cases as surrogates of infection for estimation of both the generation time and the reproduction numbers. Often though, it may be possible to characterise a distribution of the time from infection to becoming a case, say ξ*_i_* where 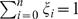. If a case is defined by symptoms then this would be the incubation period distribution. One can then back-calculate incidence as follows
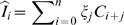
(11)


A drawback of this approach is that the estimated incidence time series *Î_i_* will tend to be over-smoothed relative to the original time series *I_i_*. It also makes clear that there is still a problem of right censoring in an incompletely observed epidemic in the estimator of equation (9), though less than in equation (10).

Statistical properties of these estimators are straightforward [12], [28]. One previously noted point [12], [28] is that because these estimators are essentially ratios of incidences, they can be used in cases where only a fraction of cases are observed, such as for polio where only a tiny fraction of infections lead to disease (of the order of 1 in 200), though the confidence intervals will change.

A special case applicable to many cases where surveillance is poor is when only the epidemic growth/decline rate *r* is known. In this case the incidence takes the form *I* (*t*) = *I* (0)exp (*rt*) and both estimators (7) and (8) for the reproduction number become
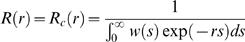
(12)where the reproduction numbers are now expressed as a function of the exponential rate of change *r*. This is likely a useful formula, presented and studied in detail in [30], where the links to earlier ecological and demographic modelling were also highlighted. Much of the subsequent analysis will concern itself with deriving an equation equivalent to (12) for the household reproduction number R^*^(*r*).

### Extending the model to heterogeneous natural histories of infection

The model defined above assumes that the function β (*t,* τ) describes the “natural history” of infection in each infected individual. Before specialising to the model of household transmission, it is first worth considering the case where different individuals experience different “natural histories”, defined here by the susceptibility to infection, and infectiousness after infection.

I denote a vector of random variables **X** = **{** *X1, X2,* …**}** to describe factors which influence susceptibility or infectiousness. For example for the standard SEIR model of infection the random variables would be the durations of the latent period (*L*) and the infectious period (*D*), i.e. **X** = **{** *L,D* **}**. Let *f* (**X** ) denote the probability distribution of these random variables amongst new infections (taking into account differences in susceptibility), defined such that
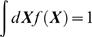
(13)where the integral is taken over the domain of the random variables. In other words, *f* (**X** ) is the proportion of new infections that have state **X**.

Let β (**X**, *t*, τ) denote the infectiousness profile of an individual with state **X**.

Assuming that all individuals mix homogeneously, then the transmission model defined earlier by equation (1) is generalised to
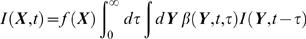
(14)where *I* (**X** , *t*) is the incidence of infections with state **X**. I define the function *K*(*t*) to denote the integral
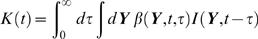
(15)which clearly depends only on time *t* and not state **X**. The total incidence at time *t* is defined by the integral
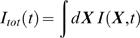
(16)


By substituting equation (14), which can be rewritten as *I* (**X**, *t* ) = *f* (**X** ) *K* (*t* ), into equation (16), I obtain that *K* (*t* ) = *I_tot_* (*t* ) and thus that
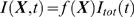
(17)


I can now substitute (17) into (14) to obtain
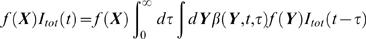
(18)


Dividing both sides of this equation by *f* **(X)**yields an equation for the total incidence
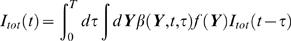
(19)


If I define the average infectiousness as follows
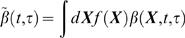
(20)then equation (19) can now be seen to be the standard Kermack-McKendrick model of equation (1), i.e.
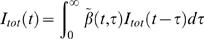
(21)


In other words, in this model of an emerging infectious disease epidemic with heterogeneities in susceptibility and infectiousness, the dynamics of mean total incidence of infection is exactly equivalent to the basic model where the infectiousness is appropriately averaged using equation (20). Once an expression is derived for the average infectiousness β̃(*t*, τ), the results such as equations (9) or (12) can be used without further consideration of the heterogeneities in infectiousness or susceptibility.

Heterogeneities which are transmitted or preserved from one infection to the next, for example due to non-random mixing between different risk groups, a situation not considered here, lead to a more complex result. Some public health interventions such as isolation and contact tracing can induce such heritability even if it is not a basic property of the transmission process [31], [32].

A useful exercise in applying this formalism (not elaborated here) is the derivation of standard formulae for the basic reproduction number as a function of the exponential growth rate *r* for the SEIR model [30].

### Estimating reproduction numbers for a model of transmission within and between households

One approach to estimating household reproduction numbers is simply to switch perspective from individual to household, directly estimate the generation time distribution (times taken for one household to infect another) and incidence of infection of households, and apply the results of equations (9) or (12) to estimate reproduction number as a function of time, R^*^(*t*), or exponential growth rate, R^*^(*r*). Because, as I have shown, the linearised Kermack-McKendrick model is applicable even when susceptibility and infectiousness are heterogeneous, this method is acceptable despite the fact that households may be quite heterogeneous in size and in the number of people infected. One analogous situation where this approach has been used is in estimating farm-to-farm reproduction numbers in the 2001 UK foot-and-mouth virus epidemic [27]. However, unless specifically tailored to this task, it is unlikely the data will be collected in the requisite form for this approach to be used in the human household situation. Thus, in this section I explore the alternative approach of explicitly modelling transmission within and between households.

**Figure 1:**
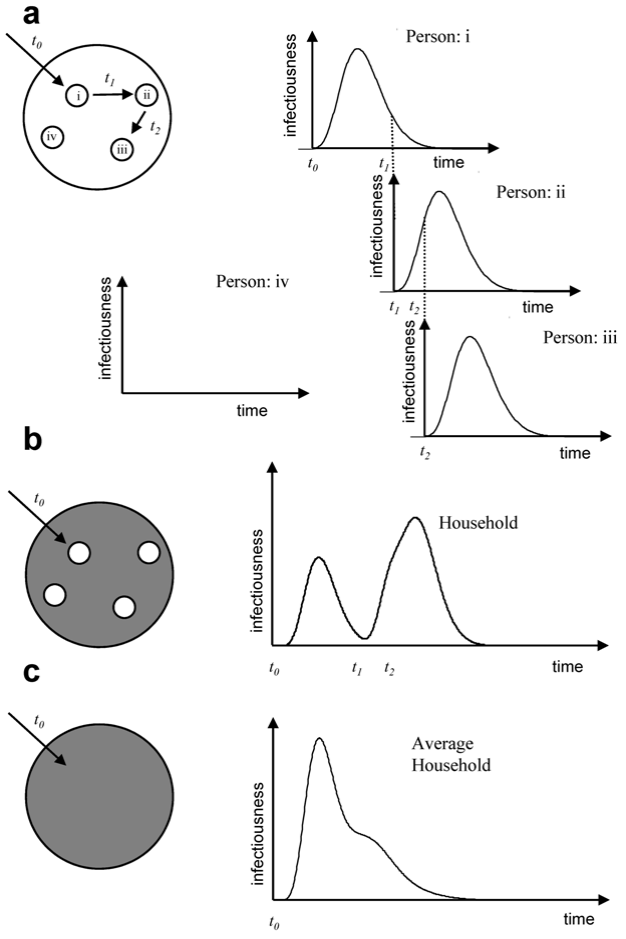
Concept of approach. As a starting point, consider a household of size 4. A, illustrates an infection being transmitted in this household, with the index case (i) being infected outside the house at time *t_0_*. They then infect exactly one person (ii) at time *t_1_*, and (ii) in turn infects person (iii) at time *t_2_*. Person (iv) escapes infection altogether. The infectiousness of each individual over time is also shown, with infection events highlighted. B, illustrates how these events can be reinterpreted by taking the unit of infection as the household, infected at time *t_0_* and with total infectiousness of the household defined as the sum of the four individual curves shown in A. The aim of the method is then to average this process over all possible chains of infection in the household, and all household sizes, to obtain the characteristic infectiousness profile of a household, as shown in C, where the household is no longer decomposed into its constituent units at all.

Homogeneous transmission models can be interpreted as two-level hierarchical models, where the processes which guide the natural history of infection within the host are considered separate from those which drive transmission between hosts. The link between the two can be thought of as the function β (*t,* τ) which translates the impact of changing processes within the host into changing infectiousness as a function of time since infection. The approach taken here to modelling household transmission is to study a three-level hierarchical model of transmission. The three levels are within-host, within-household, and between households. The natural history of infection is described by the individual infectiousness function β (*t,* τ). I assume in this section that individuals are homogenous in infectiousness and susceptibility. I then use this to predict the course of epidemics within households, and derive a function β ^*^ (*t*, τ ^*^) which describes the average infectiousness of a household towards other households as a function of the time since the household was infected, τ ^*^ (from here-on, I use the starred symbols to denote properties of households, and un-starred symbols to denote properties of individuals). The basic idea behind this analysis is illustrated in Fig 1.

To simplify the notation, and because the main aim of this section is to study the case of an epidemic growing exponentially, I consider the situation where infectiousness is independent of calendar time *t*. This could be relaxed, though only if variation in time is somewhat slower than the typical duration of infection within a household.

More specifically, the model assumptions are that:

- individuals are distributed into households, and mix randomly and homogeneously outside of their household;
- within a small time interval δ, an individual who has been infected a time τ ago infects a person at random in the population with probability β*_G_* (τ )δ;
- within this same time interval he or she infects each susceptible individual in his or her household with probability β*_L_* (τ, *n*)δ (this is allowed to depend on the household size *n*, since empirical evidence suggests such variation may occur [10]);
- the population is large, and the disease has low prevalence, so that the probability of a household being repeatedly infected is negligible;
- the functions β*_G_* (τ ) and β*_L_* (τ, *n* ) are proportional to each other as functions of the time since infection *t*.

As a result of the last assumption and of the discussion around equation (6), the infectiousness functions can be decomposed as β*_G_* (τ) = *R_G _w* (τ) and β*_n_* (*n*, τ) = *r_n_ w* (τ), where *R_G_* is the average number of people each infected individual infects through random (non-household) contacts, *w* (τ) is the generation time distribution for between household transmission, and *r_n_* is a parameter describing infection within the household whose interpretation will be clarified below.

I start by analysing the process of transmission within a single infected household of size *n* in terms of the functions *r_n_* and *w* (τ). Consider first a household of size 2, where one individual is infected at time τ ^*^ = 0. Given the Poisson process described by the assumptions listed above, the probability that the second individual remains uninfected at time τ ^*^ is 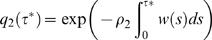. The probability that the second person is never infected is 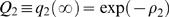. The distribution of times of infection of the second individual, conditional on infection, is then 
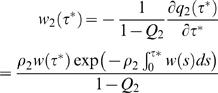
(22)where −∂ *q_2_* (τ ^*^)/∂*t* ^*^ is the rate of change of the cumulative probability of not being infected, i.e. the probability density of being infected at time τ ^*^, and the normalising factor **1-** *Q_2_* is the total probability of being infected. The difference between *w_2_* (τ ^*^) and the standard generation time distribution *w* (τ) is a saturation effect, so that the second case tends to get infected earlier as the infectiousness of the index case (ρ*_2_*) is increased.

The infectiousness of the second individual towards other non-household members of the population, conditional on his or her infection, and described as a function of the time τ ^*^ since the infection of the household is thus the convolution of *w_2_* (τ ^*^ ) and β*_G_* (τ), so that the total infectiousness of the household is 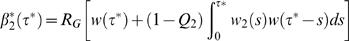
(23)


Generalising this exact result to larger households involves some complications. Consider for example a household of size 3, where one individual is infected at time τ ^*^ = 0. The probability that neither of the other two individuals is infected by the first individual at time τ ^*^ is 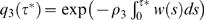 directly analogous to the situation for households of size 2. However this is somewhat greater than the actual probability that they are not infected at all, since once one of these two is infected, they can also infect the other, and thus the probability that they each escape infection is somewhat less than 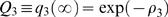.

To progress further with analysing this system, I propose to approximate the process by assuming that infections within a household can be approximately described by a discrete generation Reed-Frost model, i.e. where the probability of not being infected in each generation is (*Q_n_* )*^m^* where *m* individuals are infected in the previous generation and *Q_n_* ° exp (**−** *r_n_* ). *Q_n_* is the escape probability of each infectious-susceptible pair of individuals considered in isolation. In the formalism proposed by Ludwig, this corresponds to using infectious rank as a surrogate for infectious generation [33]. Dynamics are recovered by assuming the times between generations are described by the standard generation time distribution *w* (τ).

The ordering of infection events has no influence on the final number of individuals infected [33], and therefore this approximation will produce exact results for the final number of people infected in each household. Because of the possibility of “later” generations preceding “earlier” ones, as noted in the case of households of size 3 above, and because of ignoring the saturation effect present in equation (22) in terms of the actual generation times within households, this approximation will overestimate the time taken for individuals to become infected in the household. Because of the general form of the relation between generation time and reproduction number seen in equation (12), this will result in over-estimates of the household reproduction number R^*^ (*r*). To provide a counter-balancing under-estimate of R^*^ (*r*), I also consider an alternative approximation obtained by assuming the same total number of cases as predicted by this Reed-Frost model, but where all cases are assumed to be infected by the first index case. This is not a formal lower bound, since in the limit of infinite infectiousness within the household, all members of the household will be infected simultaneously upon introduction of the infection into the household. I find however that even for the example of highly infectious measles virus (below), the under-approximation is sufficient to provide a practical lower bound.

The probability of different chains of infection within households can easily be computed from the assumed Reed-Frost model [2]. I denote *pr*( **{** *m*_1_, *m*_2_, …, *m_n_* **}**|*n*) the probability of a chain of infection occurring in a household of size *n* where *m*_1_ index cases infects *m*_2_, who in turn infect *m*_3_ tertiary cases and so on, up to a maximum of *n* generations of infection. It is an assumption of the model that the number of index cases is 1, so *m*_1_ = 1. The full probabilities can be obtained as products of binomials linking each generation to the previous ones as follows
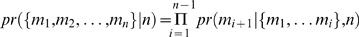
(24)where
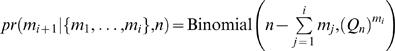
(25)


The second approximation is that the time taken for one infected to infect the next is distributed according to the standard generation time distribution *w* (τ). The time at which someone in the (*i*+1)*^th^* generation of infection is infected is as a result drawn from the *i^th^* auto-convolution of this distribution, denoted here *w* **_[_**_*i***]**_(τ ^*^) and defined by the recursive convolution equation 
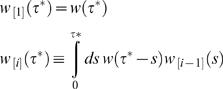
(26)which satisfies 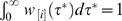.

Consider now an individual in the *i^th^* generation of infection in the household, and consider this household at a time τ ^*^ after the first index case was infected. This individual must have been infected at some earlier time *s* ⩽ τ ^*^ distributed according to the distribution *w* **_[_**_*i*-1**]**_(*s*). His or her infectiousness to others outside of the household will be given by β*_G_* (τ^*^-*s*). Thus, by averaging over all possible values of *s*, the average infectiousness of such an individual in the *i^th^* generation is 
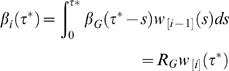
(27)


Thus having averaged over all possible times of infection in the chain of transmission events in the household, infectious households are stratified by their size and by the number of cases in each generation. Using the notation defined earlier, I define the state vector **X** = **{** *n*, *m*_1_, …, *m_n_* **}** of variables which define the infectiousness and susceptibility of *the infected household*, where *n* is the household size and *m_i_* is the number of infected individuals in the *i^th^* generation of infection in the household. The infectiousness of a household with this state **X** towards other households, mediated by random mixing of individuals between households, is the sum of the infectiousness of all the individuals each given by equation (27), i.e. 
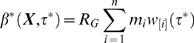
(28)


Given that this infection process involves random mixing of individuals outside their household, the distribution of sizes of households which get infected is the so-called size-biased household distribution. This is the distribution of sizes one obtains by sampling individuals at random in the population and recording the size of their household, as opposed to the more commonly recorded household size distribution which is obtained by sampling households at random. If *k_n_* denotes the household size distribution, then 
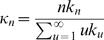
(29)is the size-biased household size distribution. Given a household of size *n* gets infected, the probability of a chain of infections is given by the Reed-Frost probabilities *pr* ({*m*_1_, …, *m_n_* **}**|*n*). The distribution of the random variables **X** = **{** *n*, *m*_1_, …, *m_n_* **}** at infection is thus
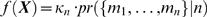
(30)


The mean infectiousness of a household is 
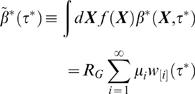
(31)where μ*_i_* is the expectation of *m_i_* under the distribution *f* (**X**), i.e. the expected number of cases in the *i^th^* generation of infection in a typical infected household given by 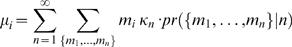
(32)


Let *μ = Σ_i_μ_i_* be the average total number of cases in an infected household. The household reproduction number takes an intuitive and well known form derived in [3], [6], expressed in terms of the parameter *R_G_* as follows: 
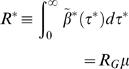
(33)i.e. the household reproduction number is the product of the expected number of infections in a household multiplied by the number of people each individual infects out of their household.

The mean household generation time distribution (time for one household infecting the next) is 
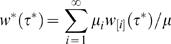
(34)


The mean generation time for households, T_g_^*^, can be expressed in terms of the individual generation time *T_g_* as 
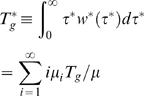
(35)


The generation time distribution w ^*^ (τ ^*^ ) can be used for the previously defined estimators of reproduction numbers (7)–(12) using household incidence data or just exponential growth rates. The exponential growth rate *r* for an exponentially growing epidemic is the same whether measured for individual or household incidence. For an exponentially growing or declining epidemic, one obtains the estimator 
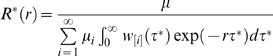
(36)


Now consider the integration 
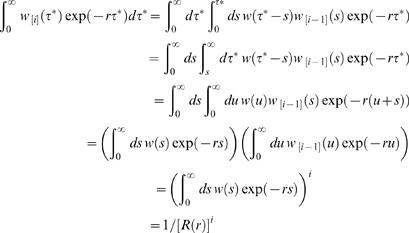
(37)where the first equality uses the definition of the auto-convolution, the second is a re-ordering of integrals, the third involves changing variables to *u* = τ ^*^-*s*, the fourth is a factorisation and the fifth arises by induction. The sixth uses the definition of the individual reproduction number *R* (*r* ) one obtains ignoring household structure from equation (12). The household reproduction number can be expressed in terms of the individual reproduction number *R* (*r* ) as 
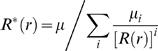
(38)


Examination of equation (33) immediately reveals that the estimate for the number of people each person infects out of the household is 
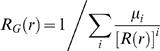
(39)


I have thus derived a simple analytic relation between the individual and household reproduction numbers. Both are approximations, ignoring the effects of local saturation on the generation time, which will tend to produce overestimates of the reproduction number. An alternative approximating to the household reproduction number, which provides an underestimate, is found when all secondary household cases are assumed to arise in the second generation, i.e. using equation (38) but substituting *μ*_1_→*μ*′_1_ = μ_1_≡1, *μ*_2_ *→μ*′_2_ = Σ^∞^ *_i = 2_μi* and *μ_i_*→*μ′_i_ = *0 for *i*>2.

## Results

### Application to influenza transmission

There are two reasons for considering household structure in analysing the pandemic influenza situation. First, influenza transmission is known to be concentrated within the household, and thus parameter estimates which ignore this heterogeneity are likely to be frail. Second, many public health policies for future pandemics are likely to be organised around the household. The net effect of social distance measures such as school and workplace closures and cancellation of social gatherings is effectively to reduce transmission out of households (and perhaps inadvertently to increase transmission within them). Furthermore, antiviral treatment and prophylaxis and quarantine measures are likely to be targeted at whole households rather than individuals (though restricting families with one suspect case to stay together without any other support is possibly undesirable) [16], [17], [20].

A number of studies have identified the parameters needed to estimate the household reproduction number for influenza [8], [10], [11], [17]. It is important to bear in mind that these parameters could be quite different in future pandemics, and thus that robust methodology may be more useful in responding to new outbreaks than numerical estimates obtained for past outbreaks. While it would be straightforward to use demographic data and exponential growth rates from earlier pandemics combined with inter-pandemic data on the transmissibility of influenza within households to obtain estimates of R ^*^ for historical pandemics, it has not been shown that the within household transmission parameters for inter-pandemic influenza adequately describe the pandemic situation, so I focus instead on providing illustrative examples using current demographic data (on the household size distribution from the UK) [34], and recent data on the transmissibility of influenza in modern households [10].

The household size data from 2001is truncated to size 6, and I assume that all households of size 6 or greater have size exactly 6. The data are *k_1_* = 29% (i.e. 29% of households are single person households), *k_2_* = 35%, *k_3_* = 16%, *k_4_* = 14%, *k_5_* = 5% and *k_6_* = 2%. The size of the mean household is thus 2.38 (average size of households where households are sampled at random), while the household of the mean individual has size 3.06 (average size of household to which individuals belong, where individuals are sampled at random).

From the French influenza study [10], I obtain maximum likelihood estimates of the within household transmission parameter of ρ*_n_* = 1.35/*n* ^1.0^ (which is consistent with the best fit to the Tecumseh data [8] of ρ*_n_* = 1.27/*n* ^0.97^). The former study followed seronegative households for a two week winter outbreak of seasonal influenza. The corresponding escape probabilities are *Q_2_* = 50.9% (i.e. the probability of not being infected by the other household member in a household of size two is 50.9%), *Q_3_* = 63.8%, *Q_4_* = 71.4%, *Q_5_* = 76.4% and *Q_6_* = 79.9%. On the scale of other infections, this places influenza as being approximately as infectious as mumps, but a lot less infectious than either varicella-zoster or measles [1]. By applying the Reed-Frost model to these data with this distribution of households, I obtain estimates of the average number of infections in each generation of infection of μ_1_ ° 1, μ*_2_* = 0.64 (i.e. the first index case directly infects an average of 0.64 people in his or her household), μ*_3_* = 0.19, μ*_4_* = 0.036, μ*_5_* = 0.0037 and μ*_6_* = 0.00021, and thus the estimate for the total expected number of cases in an infected household is *μ* = Σ^6^ *_i_*_ = 1_ *μ_i_* = 1.87, to be compared to the mean size of 3.06. These calculations are performed in Microsoft Excel 2007 using equation (25).

There is not yet a consensus on the generation time of influenza [13], [14], [16], [30], [35], with estimates ranging from 2.6 days in [13] to 5.3 days in [14]. I use a Gamma distribution with mean *T_g_* = 2.85 days and standard deviation 0.93 days, as reported in [30].

**Figure 2:**
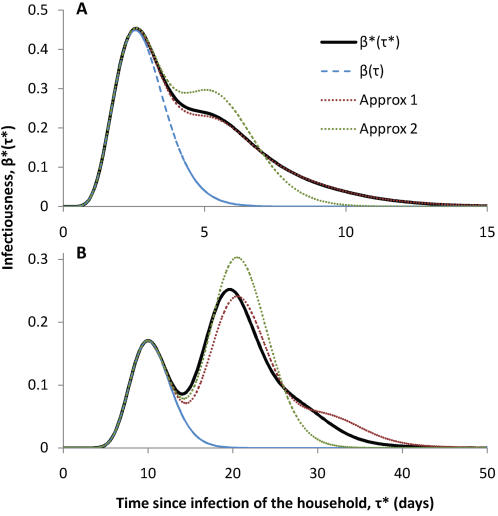
The infectiousness of households. The average infectiousness of a fully susceptible household infected with influenza (A) or measles (B). The infectiousness of individuals (denoted β (τ)) is shown, as is the infectiousness of the typical infected household (denoted β^*^ (τ^*^)). This latter curve is obtained by simulating over 10,000 epidemics of transmission within households starting from one infected case. The two analytical approximations described in the text are also shown. “Approx 1” is the main approximation described, while “Approx 2” is the one obtained by assuming that all infections occur in the second generation of infection within the household. Parameters are as described in the main text, and the curves are arbitrarily scaled such that each individual infects on average one person outside of the household (i.e. *R_G_* = 1).

Based on these data, I compare the predicted and simulated infectiousness of households in Fig. 2, which shows the average over all households sizes and compares this to the final analytical approximation given by equation (31) for β^*^ (τ ^*^), and also the alternate approximation which considers all secondary infections to arise in the second generation of infection ; the simulations and the first approximation are clearly in good agreement. Individual based stochastic simulations were programmed using Berkeley Madonna, and are described in Appendix S1.

**Figure 3:**
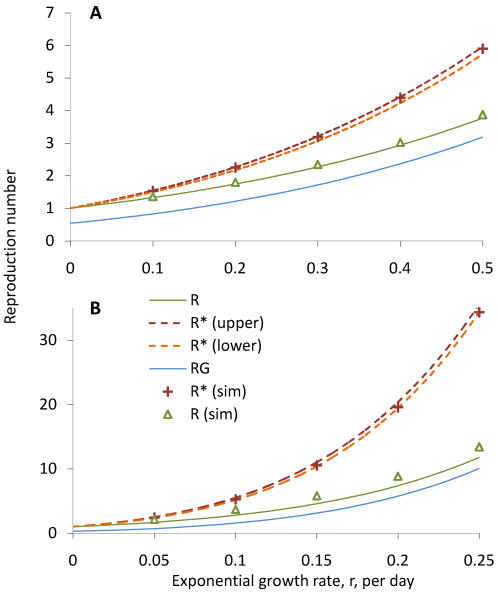
Reproduction numbers for an exponentially growing epidemic. Estimates of the individual reproduction number *R* (*r* ) (the average number of people infected by each individual) and the household reproduction number R^*^ (*r* ) (the average number of people infected by each household) are shown as a function of the epidemic growth rate *r* for influenza (A) and measles (B). The two analytical approximations which bracket the household reproduction number R^*^ (denoted upper and lower) are shown along with numerically estimated values (‘+’ symbols). The approximation to the individual reproduction number given by equation (12) is shown (dashed line) along with numerical estimates (‘Δ’ symbols). The estimate for the quantity *R_G_*, the average number of people each person infects outside his or her home, is also shown.

For the case of an exponentially growing epidemic, the estimates of the individual and household reproduction numbers, *R* and R ^*^ respectively, are shown in Figure 3, along with the estimate of the number of people one person infects outside their household, *R_G_*. For R ^*^, both the under- and over-estimating approximations are shown, along with estimates obtained from the simulated generation time distribution. As expected for this low-infectiousness scenario, the simulated values are closer to the over-estimating approximation. The range between these approximations which bracket the true value is rather narrow, indicating that the method is predictive.

For the 1918 “Spanish Flu” H1N1 pandemic, the median growth rate in large US cities was *r* = 0.20 per day [30], [35], with comparable estimates in the UK [17]. This value also serves as an upper estimate for the spread of the H2N2 pandemic virus in 1957 [17]. Based on this growth rate, the estimated individual reproduction number is *R* = 1.74, while the estimated household reproduction number is R ^*^ = 2.26, and thus the out-of-household reproduction number is *R_G_* = 1.21. Of course, households were bigger in 1918 than now, so that the actual value of R^*^ was likely higher than this. These estimates would imply that a proportion 1−1/R ^*^ = 56% of between household transmission would need to be blocked to prevent epidemic spread. Figure 3 could provide a rough guide to the likely values of R^*^ and *R_G_* for a new influenza pandemic where the rate of exponential growth can reliably be determined.

Consider someone who the index case in their household; they would be expected to infect *R_G_* = 1.21 people out of their household and μ*_2_* = 0.64 within their household. This validates assumed proportions of transmission within and between households from earlier simulation studies [17], [20]. The sum of these is greater than *R* since the reproduction number *R* is an average over different generations of infection within the household. For this value, the estimate of *R* which takes into account local saturation effects was determined numerically to be *R* = 1.79. Fig 3 shows that for all values of *r*, numerically estimated values for *R* (*r* ) are close to the curve estimated from application of equation (12) which ignores local saturation effects.

**Figure 4:**
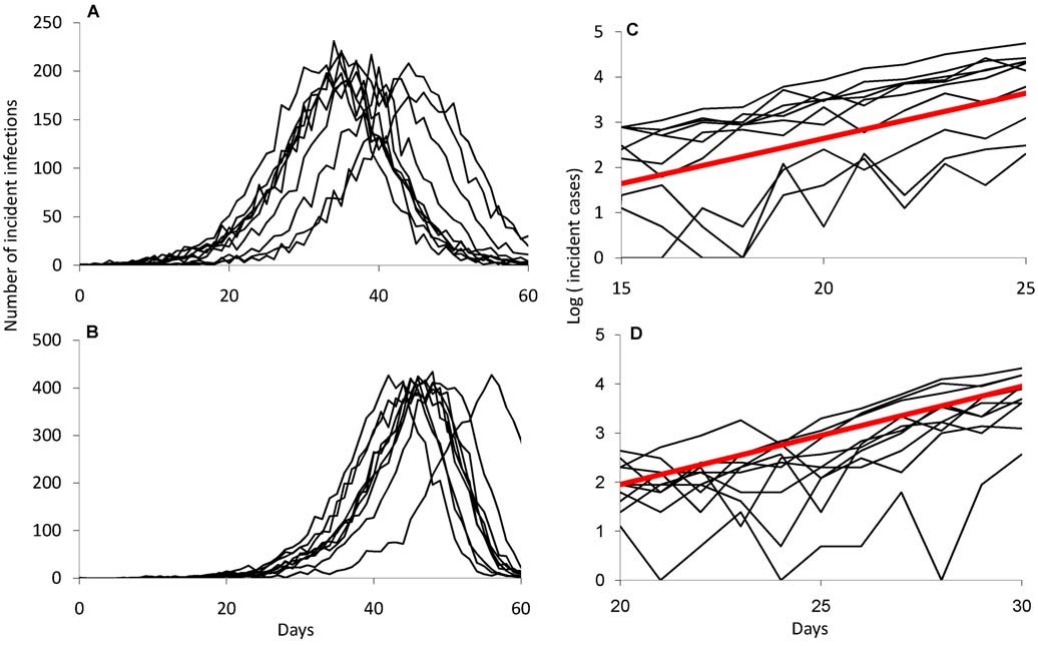
Simulated epidemics. To check the method for consistency, I simulate ten epidemics of influenza (A) and measles (B) within a fully susceptible community of 2,000 households. I use parameters estimated for an epidemic growth rate *r* = 0.20 per day, and condition on non-extinction of the epidemic. In C and D, the natural logarithm of the incidence is compared to the fixed slope curve *r* = 0.20 predicted by the model (thick line). Linear regression through these data yields the estimate *r̂* = 0.19 in both cases.

As a final check of the method, epidemics within a community of 2,000 households were simulated using an individual based stochastic model (see Appendix S1). I choose *R_G_* = 1.21 as inferred from an epidemic growth rate of r = 0.20 per day, and the other parameters as described above. The exponential rate of growth was then re-estimated directly from the simulated incidence time-series to be r = 0.19 (Figure 4), close to the predicted value of r = 0.20. This provides further support for the validity of this method, especially since no restrictions were placed on re-infection of households within this small simulated community.

### Application to measles transmission

As noted above, influenza is relatively uninfectious compared to other common viruses. For a contrasting application of the method, I now focus on measles which was the most infectious of the pathogens studied in [1]. Measles also has a more peaked generation time distribution, so that generations of infection are more distinct, and to make the contrast with the influenza estimates yet greater, I also use demographic data on household size chosen from the national census in 1961, when household sizes were greater than they are now. This analysis is perhaps a little artificial when applied to measles, since a large proportion of the population will have immunity either due to past infection or vaccination with the live MMR vaccine. The principal motivation is to further test and illustrate the methods in a case where good data on the transmission dynamics within households are available. Stratification by household of the recent outbreaks of measles caused by decreasing uptake of the MMR vaccine could reveal whether household heterogeneities should have be accounted for in estimating the changing reproduction number of measles [36].

The household size data from 1961 is truncated to size 6, and I assume that all households of size 6 or greater have size exactly 6. The data are *k_1_* = 14% (i.e. 14% of households are single person households), *k_2_* = 30%, *k_3_* = 23%, *k_4_* = 18%, *k_5_* = 9% and *k_6_* = 7%. The size of the mean household is thus 2.99 (average size of households where households are sampled at random), while the household of the mean individual has size 3.66 (average size of household to which individuals belong, where individuals are sampled at random).

Hope-Simpson reported susceptible-infectious escape probabilities of *Q* = 69.9% for mumps, *Q* = 39% for varicella, and *Q* = 24.4% for measles in under 15s [1]. The results were reported independent of household size, and were regarded as unreliable in over-15s. Based on applying the Reed-Frost model to the measles estimate with this distribution of households, I obtain estimates of the average number of infections in each generation of infection of μ*_1_* ° 1, μ*_2_* = 2.01 (i.e. the first index case directly infects an average of 2.01 people in his or her household), μ*_3_* = 0.50, μ*_4_* = 0.020, μ*_5_* = 0.00036 and μ*_6_* = 0.0000031, and thus the estimate for the total expected number of cases in an infected household is *μ* = Σ^6^ *_i = _*_1_ *μ_i_* = 3.54, to be compared to the mean size of 3.66.

Hope-Simpson also reported the intervals between linked cases in households using different case definitions [1]; the intervals for what he regarded as the most reliable case definition, “maximum rash”. These data is well described by a Gamma distribution (not shown). The maximum likelihood estimate of the generation time is *T_g_* = 10.5 days with standard deviation 2.4 days.

Based on these data, I repeat the simulations of the previous section on influenza but with parameters for measles in Figs 2, 3 and 4. Figure 2B shows that, as expected, the average infectiousness of a household is less well approximated by either approximation than for the much less infectious case of influenza. In this case, multiple peaks of infectiousness corresponding to generations of infection within the household can be clearly distinguished, and there are more cases in the second generation of infection than in the first.

In terms of the predicting of the household reproduction number R^*^, the method is still found to be strongly predictive (as evidence by the small gap between upper and lower estimate) and reliable (compared to numerical estimates). While in influenza, the simulations were close to the upper approximation, here they are closer to the lower approximation, as expected for the more infectious situation of measles transmission. Simulations of transmission within a community of households were again found in Figure 4B to validate the approach. The difference in the shape of the epidemic curve with influenza reflects the different shape of the generation time distribution, though the exponential growth rate is the same.

## Discussion

New methods were presented to estimate both the individual and household reproduction number during an epidemic. The new method presented for estimating the individual reproduction number relates closely to earlier work [12], [27], [30], but provides an alternative and possibly simpler solution to the problem of incomplete observations during an unfolding epidemic [29]. It also provides an alternative and perhaps more satisfying solution than the incidence-to-prevalence ratio method [37], [38] to the problem of long generation time distribution infections such as HIV, where epidemiological circumstances can change substantially within the course of a single infection, and thus the case reproduction number represents too much of an average to convey secular changes in behaviour and transmission.

Nothing in this study challenges the central role of the individual reproduction number as an epidemiological measure; because the empirical measures of reproduction number proposed here and in [12], [27], [29] use incident observed cases as the base, all of the complication in defining the ‘typical’ or ‘eigen’ case for structured models discussed most clearly in [24] are neatly sidestepped. What this study does highlight is that much complexity is hidden in effectively defining and estimating the generation time distribution for a structured population. In the case studied here, generation times between individuals are shorter for within household transmission than between household transmission, particularly for more infectious pathogens, and this resulted in systematic biases associated with estimating the reproduction number while ignoring this effect, which were quite substantial in the case of highly infectious measles virus.

The methods presented for the estimation of household reproduction numbers were not affected by this problem in the same way. Analytical approximation were derived which bracketed estimates between a lower and upper bound, and numerical simulations showed the range within these brackets to be narrow. These approximations were shown to be robust, but it is worth noting that assumptions are made about the population mixing randomly out of their households and results are only valid in the scenario of an emerging pathogen where overall prevalence is low.

The usefulness of these methods is likely to be found in predicting and understanding the impact of household targeted infection control measures in an emerging epidemic. This actually covers a wide class of interventions since the household is a central living and administrative unit in most populations. Decisions regarding isolation, quarantine, vaccination and prophylaxis may often be made for entire households. Similarly school and workplace closures as well as restrictions on leisure activity can be thought of as trying to reduce between household transmission. Analytical approaches are also invaluable in calibrating and providing independent checks on more detailed individual based micro-simulations, such as [13], [17], [20]. Some control interventions require more subtle analyses; for example it has been shown that vaccinating whole households is not the most effective strategy for a given vaccine coverage rate, and that alternative strategies such as preferentially vaccinating larger households could be considered [39].

Further avenues of research include studying the statistical properties of these estimators for different situations. The assumption made here, that individuals mix nearly homogeneously out of their household may be an appropriate approximation for describing transmission within a neighbourhood or even a city [20], but ultimately one should also consider developing the estimators for more complex demographic situations such as a hierarchy of organisations (household, to village, to region, to country, etc…) or a more complex overlap of households, workplaces and regular social spaces. Also of interest is the study of intervention measures, particularly those that respond to the presence of a symptomatic cases; the measures of pre-symptomatic transmission presented in [25] clearly generalise to a household, but analytical results on the efficacy of isolation and quarantine are not evidently obtainable.

The estimators of the household reproduction number have been shown here to be robust on their own terms, but I have not addressed the issue of model misspecification, for example to inaccurate determination of the generation time distribution or to individual heterogeneity in infectiousness or susceptibility within households. Further scenarios could be explored both to test the method with different infections and to address the issue of model misspecification.

There are many cases where it may be desirable to quantify household transmission, but where a degree of natural or vaccine-induced immunity may be present in the population, a problem not addressed here. In considering these more complex situations, while it may not be possible to obtain analytic forms for the infectiousness of a household, numerical forms can usually be obtained quickly and still offer benefits over full individual based micro-simulations in easily exploring a wide range of parameters.

Finally, the likely practical benefits of estimating household transmission parameters in an emerging epidemic need to be clearly established and communicated, and the most effective ways to enhance data collection protocols to allow their rapid estimation need to be identified.

## Supporting Information

Appendix S1 Description of the simulations
(0.08 MB PDF)
