# Supplementary material for: Estimating Individual and Household Reproduction Numbers in an Emerging Epidemic
Source: PLoS One. 2007 Aug 22;2(8):e758. doi: 10.1371/journal.pone.0000758 (PMC1950082; doi:10.1371/journal.pone.0000758)
Supplement: Appendix S1 — Description of the simulations (0.08 MB PDF) [file pone.0000758.s001.pdf]

Estimating Individual and Household Reproduction Numbers in an Emerging Epidemic

Christophe Fraser

MRC Centre for Outbreak Analysis and Modelling, Department of Infectious Disease Epidemiology, Imperial College London, UK.

c.fraser@imperial.ac.uk

Appendix S1: Description of the simulations

Simulating Transmission in a Typical Household

To simulate transmission within a single household for numerical determination of $\beta^*\left(\tau^*\right)$ and R^*, a household size n was randomly drawn from the size biased household size distribution κ_n. Each individual was labelled $i=\{1,...,n\}$ and was allocated an infected-status indicator I_i and time-since-infection τ_i. Uninfected susceptible individuals have $I_i=0$ while infected individuals have $I_i=1$. Time-since infection is frozen to $\tau_i=0$ for all uninfected individuals.

At time $\tau^*=0$, individual 1 is infected from outside the household.

At each time-step, individuals are infected with probability $\Lambda=\delta\sum_{i=1}^{n} I_i\beta_L\left(n,\tau_i\right)$ where δ is the time-step size. The infectiousness of the household is given by $\beta^*\left(\tau^*\right)=\sum_{i=1}^{n} I_i\beta_G\left(\tau_i\right)$. After each time-step, time-since-infection is incremented by $\tau^*\to\tau^*+\delta$ and $\tau_i\to\tau_i+\delta$ for all i such that $I_i=1$.

10,000 simulations were carried out for Figures 2 and 3 with time-step 0.02 for influenza and 0.05 for measles.

Simulating Transmission in a Community of Households

To simulate transmission within a community of M households, M household sizes n_α were randomly drawn from the standard household size distribution k_n, where $\alpha = 1,...,M$ labels the households. Each individual was labelled (i,α) where $i = \{1,...,n_\alpha\}$ and was allocated an infected-status indicator $I_{i,\alpha}$ and time-since-infection $\tau_{i,\alpha}$.

At time $t = 0$, individual $(1,1)$ is infected from outside the community.

At each time-step, individuals within each household α are infected with probability $\Lambda_\alpha = \delta\Big(\sum_{i=1}^{n_a} I_{i,\alpha}\beta_L(n,\tau_{i,\alpha}) + (1/N)\sum_{\beta=1}^{M}\sum_{i=1}^{n_\beta} I_{i,\beta}\beta_G(\tau_{i,\beta})\Big)$ where the first term represents transmission within households and the second represents random mixing, $N = \sum_{\alpha=1}^{M} n_\alpha$ is the total population size and δ is the time-step size. After each time-step, time is incremented by $t \to t + \delta$ and time-since-infection is incremented by $\tau_{i,\alpha} \to \tau_{i,\alpha} + \delta$ for all $i - \alpha$ such that $I_{i,\alpha} = 1$.

10 simulations in populations of $M = 2,000$ households were carried out for Figure 4 with time-step 0.02 for influenza and 0.05 for measles.
